# Is glycoprotein VI involved in contractual negotiations?

**DOI:** 10.1016/j.rpth.2024.102329

**Published:** 2024-01-26

**Authors:** Simone A. Brysland, James I. Hearn, Elizabeth E. Gardiner

**Affiliations:** Division of Genome Science and Cancer, John Curtin School of Medical Research, Australian National University, Canberra, Australia

Glycoprotein (GP) VI is the primary signaling receptor for collagen and is found exclusively on platelets and megakaryocytes. GPVI engagement by exposed collagen within a damaged vascular space initiates thrombus formation, and subsequent GPVI interactions with fibrin contribute to thrombus growth and stability [1]. While the GPVI signaling pathway that uses an immunoreceptor tyrosine–based activation motif (ITAM) present within the cytoplasmic tail of the linked FcRγ chain is well characterized [2], how GPVI activation modulates the platelet cytoskeleton remains an open question. In this issue of *Research and Practice in Thrombosis and Haemostasis*, Kenny and colleagues [3] investigated how alterations in the activity of the cytoskeletal protein myosin in platelets impacted the subcellular architecture of the platelet cytoskeleton and platelet functional outcomes.

Cellular contractility is driven by actin filaments interacting with myosin in a process known as motor stepping. Myosin binds to filamentous actin and undergoes cyclic conformational changes fueled by the hydrolysis of adenosine triphosphate (ATP) to exert a pulling force on actin. Nonmuscle myosin II is the major myosin isoform in megakaryocytes and platelets and contributes to the cytoskeletal dynamics that underlie the contractile force required for thrombus and clot retraction as part of a normal hemostatic response. Mutations or inhibition of myosin IIA result in macrothrombocytopenia as well as defective contractility and bleeding [4,5]. Roles for the GPIb-IX-V complex, α_IIb_β_3_, and von Willebrand factor (VWF) in mediating these contractility forces have been well described [6]. Here, the authors examined human platelet behaviors using τ-stimulated emission depletion microscopy and blebbistatin (BBT), an inhibitor with specificity for myosin IIA. The goal was to measure traction force and assess intraplatelet nanoarchitecture. Experiments examining roles for myosin in platelet function at different stages of thrombus formation uncovered an unexpected role for myosin IIA in GPVI-mediated pathways.

The authors identified that mild myosin inhibition achieved by careful dosing of BBT altered platelet stress fiber formation (contractile actin bundles) by reducing the generation of platelet traction forces and, thus, reducing highly contractile platelet numbers (see Figure). This was explained by the presence of actin binding–competent but stepping-incompetent myosin motors, which disturbed collective stepping motions. Under these conditions, platelet adhesion to VWF under flow, as well as the spreading area and cytoskeletal rearrangements on fibrinogen, were not impacted, suggesting that even limited myosin activity was sufficient to support these processes. Additionally, complete myosin inhibition reduced platelet adhesion to fibrinogen under flow, possibly by altering lamellipodia formation and abolishing platelet traction forces. Of the platelets that did adhere, platelet spreading, α granule release, and procoagulant phosphatidylserine exposure were unaffected.

**Figure:**
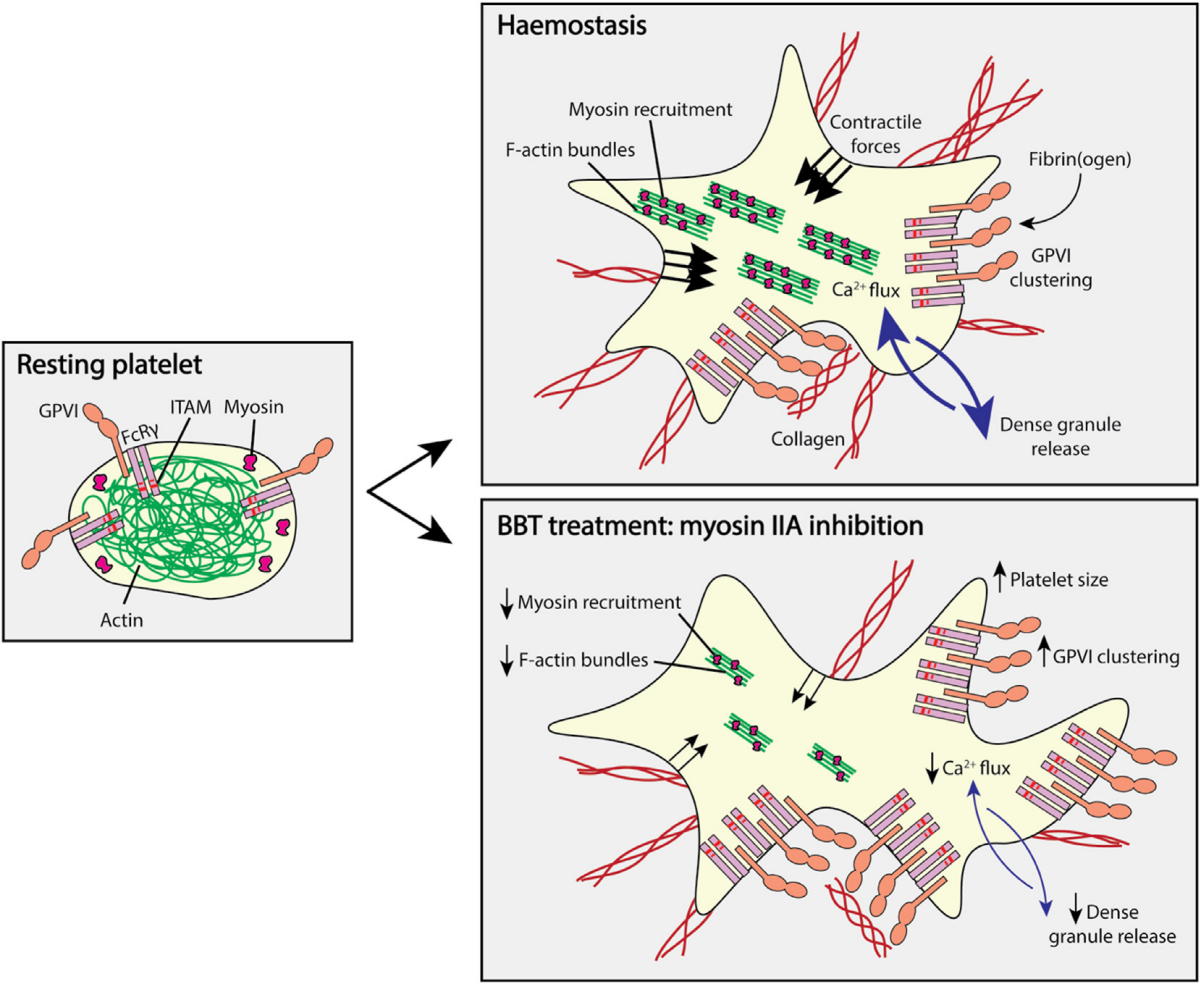
Myosin IIA inhibition results in increased GPVI clustering and reduced GPVI-mediated platelet activation. Treatment of platelets with BBT, an inhibitor of myosin IIA activity, results in altered platelet function. BBT impairs cytoskeletal dynamics with reduced F-actin bundle formation and myosin recruitment. BBT also results in increased clustering of GPVI, with reduced platelet Ca^2+^ flux, GPVI-mediated platelet activation, and dense granule release. BBT, blebbistatin; GP, glycoprotein; ITAM, immunoreceptor tyrosine–based activation motif.

The authors showed that myosin inhibition increased GPVI clustering and reduced Ca^2+^ flux, GPVI-mediated platelet activation, and dense granule release. It will be of interest to assess whether this represents impaired receptor-mediated Ca^2+^ entry or a systemic disruption of platelet Ca^2+^ homeostasis. Beyond the molecular motor features of myosin IIA, this protein played an important role in contracting and cross-linking the platelet cytoskeleton, with evidence of a supportive role in GPVI signaling. Aligning with this notion, myosin IIA inhibition has previously been shown to reduce FcγRIIa-mediated platelet spreading on heat-aggregated immunoglobulin G (IgG) [7] and FcRγ ITAM phosphorylation and Syk recruitment in macrophages in response to IgG-opsonized beads [8]. The authors speculated that coregulation of GPVI signaling by myosin could be indirectly controlled by the ATP-gated P2X1 cation channel. In platelets, P2X1 is activated by ATP released from dense granules, enhancing Ca^2+^ flux, and is known to synergize with GPVI to amplify responses to agonists present in low abundance [9]. Evidence that myosin associates with P2X7 in monocyte (THP-1) and kidney (HEK-293) cell lines [10] supports the rationale that myosin can interact with P2X1; however, this interaction remains to be demonstrated in platelets.

One aspect that remains unclear from the work of Kenny et al. [3] is an explanation of precisely how GPVI function is influenced by components of the platelet cytoskeleton. The human GPVI cytoplasmic tail encompasses 51 amino acid residues and contains binding sites for calmodulin, tyrosine kinases, and adaptor proteins such as tumor necrosis factor receptor–associated factor that link GPVI with redox systems to generate reactive oxygen species [11]. However, evidence of direct molecular links between GPVI and myosin or other platelet cytoskeletal components is lacking. Similar to GPVI coopting the services of the FcRγ chain to utilize ITAM signaling, GPVI may also form an alliance with other platelet receptors that are actively engaged with the platelet cytoskeleton. The GPIb-IX-V complex is one potential candidate here for several reasons. First, GPIb-IX-V is abundantly and, like GPVI, specifically expressed on platelets and has clear associations with cytoskeletal filamin, actin, and actin-binding proteins [12]. Second, the ectodomains of GPIbα and GPVI have been shown to interact noncovalently on the platelet surface and coimmunoprecipitate [13]. Third, the GPVI response to ligands and, thus, function can be modulated by monoclonal antibodies targeting specific regions of the GPIbα subunit of GPIb-IX-V [13,14]. Fourth, optimal GPVI-mediated ITAM signaling was shown to require an intact GPIbα intracellular tail and filamin since transgenic mice deficient in filamin A or expressing a truncated form of GPIbα that was missing the last 24 residues of the cytoplasmic tail, mounted attenuated responses to GPVI agonists [15,16]. Therefore, GPVI may be indirectly associated with the platelet cytoskeleton through its interactions with GPIbα. While there is no evidence that filamin and myosin directly cointeract, these 2 operators both bind to actin and share common signaling pathways involving Rho GTPases [12].

Using STochastic Optical Reconstruction Microscopy (STORM) super-resolution microscopy, the authors noted increased lamellipodia-localized GPVI clustering on collagen with myosin IIA inhibition despite decreases in GPVI-mediated platelet activation responses. Enhanced GPVI clustering has been shown to result in increased GPVI signaling [17], so the current finding underscores the notion that myosin IIA coregulates GPVI downstream signaling pathways independent of clustering. Previous studies have shown that actin antagonism reduced GPVI clustering [17], suggesting that actin and myosin play distinct roles in guiding GPVI motility within the platelet plasma membrane. It will be intriguing to understand more deeply how the GPVI-myosin alliance guides Rho GTPase activity and thrombus contraction, as incorrect functioning of this pathway is likely to enhance bleeding tendencies in mice [18] and in humans [19]. We eagerly await future studies that aim to formally test these hypotheses using a range of other inhibitors and evaluate contributions of the P2X1 receptor and the GPIbα subunit.
